# Efficacy of products for bleaching and whitening under orthodontic brackets

**DOI:** 10.1590/2177-6709.27.5.e2220325.oar

**Published:** 2022-11-07

**Authors:** Célia Regina Maio PINZAN-VERCELINO, Suellen Nogueira Linares LIMA, Fernando Félix de Jesus Vieira PEREIRA, Júlio de Araújo GURGEL, Gisele Rodrigues da SILVA, Karina Maria Salvatore de FREITAS

**Affiliations:** 1 Centro Universitário Uningá, Departamento de Odontologia (Maringá/PR, Brazil). cepinzan@hotmail.com; 2 Universidade Ceuma, Departamento de Odontologia (São Luís/MA, Brazil).; 3 Universidade Estadual de São Paulo (UNESP) (Marília/SP, Brazil).; 4 Universidade Federal de Uberlândia, Escola de Odontologia, Departamento de Dentística Operatória e Materiais Odontológicos (Uberlândia/MG, Brazil).; 5 Centro Universitário Uningá, Departamento de Odontologia (Maringá/PR, Brazil).

**Keywords:** Tooth bleaching, Toothpastes, Tooth bleaching agents, Orthodontic brackets

## Abstract

**Introduction:**

Many patients wearing orthodontic appliances request alterations in the shade of their teeth during orthodontic treatment.

**Objective:**

This study aimed to evaluate the efficacy of different products for bleaching and whitening under orthodontic brackets.

**Methods:**

Seventy bovine incisors were randomly divided into five groups (n = 14): C) non-whitening toothpaste (control); WTsi) hydrated silica whitening toothpaste; WThp) 2% hydrogen peroxide whitening toothpaste; OB) in-office bleaching; and HB) at-home bleaching. Two buccal surface areas were evaluated using the Easyshade spectrophotometer: under the metal bracket (experimental) and around the bracket (control). The paired *t*-test, ANOVA, and Tukey tests were applied for statistical analysis.

**Results:**

Intragroup comparisons showed that in groups C, WThp and HB, there were statistically significant differences in the enamel color changes (ΔE_ab_) between under and around the bracket areas (C - under bracket = 7.97 ± 2.35, around bracket = 2.86 ± 0.81, *p*< 0.01; WThp - under bracket = 4.69 ± 2.98, around bracket = 2.05 ± 1.41, *p*< 0.01; HB - under bracket = 7.41 ± 2.89, around bracket: 9.86 ± 3.32, *p*= 0.02). Groups WTsi, OB and HB presented similar perception of tooth whiteness (ΔWI_D_) between the tested areas. Intergroup comparisons demonstrated that under the bracket area, the color change (ΔE_ab_) was similar for all groups, except WThp (C = 7.97 ± 2.35; WTsi = 8.54 ± 3.63; WThp = 4.69 ± 2.98; OB = 9.31 ± 4.32; HB = 7.41 ± 2.89; *p*< 0.01).

**Conclusions:**

The dental color changes were effective for the products tested in groups WTsi, OB and HB in the presence of metallic orthodontic brackets.

## RESUMO

**Introdução:**

Durante o tratamento ortodôntico, muitos pacientes solicitam, também, alteração na cor dos seus dentes.

**Objetivo:**

O presente estudo avaliou diferentes produtos clareadores e branqueadores, quanto à sua eficácia sob os braquetes ortodônticos.

**Métodos:**

70 incisivos bovinos foram aleatoriamente divididos em cinco grupos (n = 14): C) dentifrício não branqueador (controle); WTsi) dentifrício branqueador com sílica hidratada; WThp) dentifrício branqueador com peróxido de hidrogênio a 2%; OB) clareamento no consultório e HB) clareamento caseiro. Usando um espectrofotômetro Easyshade, foram avaliadas duas áreas na superfície vestibular dos dentes: sob o braquete (experimental) e ao redor do braquete (controle). Para análise estatística, foram usados os testes*t* pareado, ANOVA e Tukey.

**Resultados:**

As comparações intragrupos demonstraram que, nos grupos C, WThp e HB, ocorreram diferenças estatisticamente significativas nas alterações de cor do esmalte (ΔE_ab_) entre as áreas sob o braquete e ao redor do braquete (C: sob o braquete = 7,97 ± 2,35, ao redor do braquete = 2,86 ± 0,81, *p*< 0,01; WThp: sob o braquete = 4,69 ± 2,98, ao redor do braquete = 2,05 ± 1,41, *p*< 0,01; HB: sob o braquete = 7,41 ± 2,89, ao redor do braquete = 9,86 ± 3,32, *p*= 0,02). Os grupos WTsi, OB e HB demonstraram semelhanças na percepção do clareamento (ΔWI_D_) entre as áreas avaliadas. As comparações intergrupos demonstraram que, nas áreas sob os braquetes, as alterações de cor (ΔE_ab_) foram semelhantes para os grupos, com exceção do WThp (C = 7,97 ± 2,35; WTsi = 8,54 ± 3,63; WThp = 4,69 ± 2,98; OB = 9,31 ± 4,32; HB = 7,41 ± 2,89; *p*< 0,01).

**Conclusões:**

As alterações na cor dos dentes foram efetivas para os procedimentos testados nos grupos WTsi, OB e HB na presença de braquetes metálicos ortodônticos.

## INTRODUCTION

Dental bleaching and alignment are the majority of complaints from patients who attend dental offices.^1^ ^-^ ^3^ Besides the aesthetic benefits, dental color and position changes have also been correlated with individuals’ social perception, including more positive judgments related to social competence and appeal, intellectual ability, and relationship satisfaction.^1^ ^,^ ^4^

Many orthodontic patients request dental bleaching during orthodontic treatment,^5^ to simultaneously achieve the desired dental color and position. Usually, dissatisfaction with the color of teeth increases when the crowding is solved.^2^

Dental bleaching and whitening are different processes. Dental bleaching involves the application of gels containing hydrogen peroxide or carbamide peroxide on the tooth surface. The peroxide diffuses through the enamel and dentin, producing free radicals that react with the intrinsic pigments, making the teeth appear whiter.^6^ ^,^ ^7^ A recent study suggested that hydrogen peroxide might whiten normal dentin by oxidizing the benzene ring of aromatic amino acids in dentin phosphoprotein, the main noncollagenous protein located in the organic-inorganic interface, responsible for the fluorescence and color of normal dentin.^8^ On the other hand, dental whitening consists of the removal and control of extrinsic tooth stains.^7^ Currently, many whitening products, with the promise of rapid and convenient color alteration, are available on the market, including whitening toothpastes.^7^ ^,^ ^9^ ^,^ ^10^ Whitening toothpastes have become popular due to low costs, unrestricted selling, ease of use, and high availability.^7^ ^,^ ^11^

Most whitening products act in one of these mechanisms: chemical (the use of peroxides for tooth bleaching) or mechanical (removal of extrinsic stains through abrasive action).^7^ ^,^ ^12^ Toothpastes that act using a mechanical mechanism contain abrasives that only remove the extrinsic stains, instead of changing the tooth color, as is observed in a real bleaching action. Some whitening toothpastes have included peroxide in their formulations; however, the efficacy of peroxide from toothpaste is questioned regarding the concentration and exposure time during toothbrushing.^13^

Previous clinical^14^ ^,^ ^15^ and laboratory^16^ ^-^ ^19^ studies have shown color alteration under the bracket area using bleaching agents. However, even with the increasing presence of whitening toothpastes on the market, the authors are unaware of a study that has been conducted to test their efficacy on teeth with bonded brackets. Therefore, the present study aimed to evaluate different products’ efficacy for bleaching and whitening under orthodontic brackets. The null hypothesis tested was that there would be no differences in color change when hydrated silica whitening toothpaste; 2% hydrogen peroxide whitening toothpaste; in-office dental bleaching using 35% hydrogen peroxide; and at-home bleaching using 22% carbamide peroxide procedures are applied on teeth with bonded brackets.

## MATERIAL AND METHODS

The local Animal Ethics Research Committee (CEUA-UNICEUMA, São Luís/MA, Brazil) approved this *in vitro* investigation (protocol 0029016). The sample size calculation was based on data previously described,^15^ ^,^ ^20^ using the dental color change (ΔE_ab_) as a reference variable. The parameters used were: 95% confidence level, power of 80%, standard deviation of 2.3 ΔE_ab_, and a minimum difference of 2.7 ΔE_ab_ between the means (G*Power software, version 3.1.3; Franz Faul, University of Kiel, Kiel, Schleswig-Holstein, Germany). The minimum number of specimens was determined to be 12 per group, and 20% was added to this value to increase the power, thus resulting in 14 teeth per group. 

Seventy experimental units were obtained from bovine incisors aged between 24 and 30 months, and were stored in a refrigerated 1% chloramine-T solution, ph7, for 30 days. The following criteria for tooth selection were used: intact buccal enamel, with no cracks, no abrasion, or any other crown defect, and similar size and shade (A3, determined by comparison with a value-oriented shade guide - Vita Toothguide 3D Master; VITA Zahnfabrik, Bad Säckingen, Bade-Württemberg, Germany). The morphological enamel conditions were evaluated by two researchers, using a simple magnifying glass. Only in cases of agreement, the teeth were selected. The researchers inspected 120 teeth and selected 70 from this set. The roots and pulp chamber of the teeth were removed. The pulp chamber was accessed and extended using a conical diamond tip. The dental pulp and debris were removed using a dental curette and air/water jets. Afterward, the bovine incisors were stored for 7 days in distilled water. 

**Figure 1:**
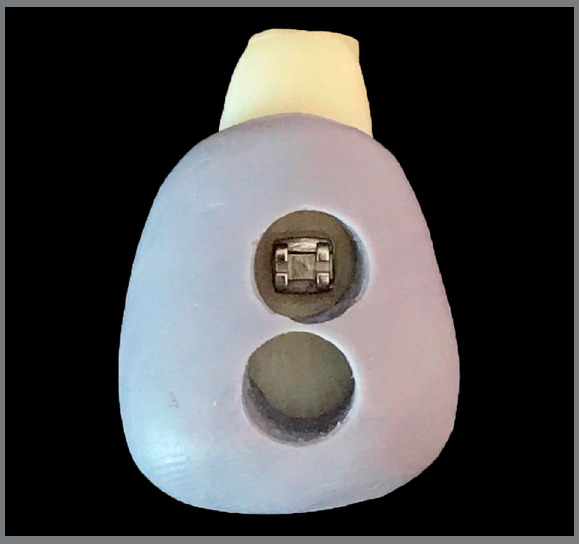
The silicone matrix made individually for each tooth, to standardize the color measurement area and guide the brackets bonding.

One matrix was made for each tooth, using high-viscosity silicone, to standardize the color measurement area, and guide the bracket bonding. In the matrix, two vertically adjacent circular windows were located close to the center of the crown, on the buccal surface of the teeth (Fig 1): one experimental area (under the bracket) and one control area (around the bracket). They were configured using a circular metal-cutting device measuring 6 mm in diameter (Biopsy Punch, Miltex; York, PA, USA), corresponding to the diameter of the spectrophotometer probe (Easyshade Advance 4.0; Vident, Brea, CA, USA).^14^ ^,^ ^15^ ^,^ ^21^ ^,^ ^22^

A single calibrated operator performed the bonding procedure. Before brackets bonding, the teeth received dental screening and dental prophylaxis, using pumice and a rubber cup with a low-speed handpiece. With the matrix installed on the buccal surface of the teeth, at the circular opening of the experimental area, 37% phosphoric acid gel was applied for 30s, followed by rinsing for 30s, and drying for 15s. The Transbond XT primer (3M Unitek Orthodontic Products, Monrovia, CA, USA) was applied, followed by a brief air spray and light-curing for 20s. Metal brackets (maxillary central incisor, Standard Edgewise, 0.022 x 0.030-in slot; Morelli, Sorocaba/SP, Brazil) were bonded on the prepared surface using Transbond XT light-cure composite resin (3M Unitek Orthodontic Products, Monrovia, CA, USA). The brackets were positioned at the center of the experimental area, and held down with 300g force for 10s, using a tensiometer. Before light-activation, the matrix was removed, and the excess material was removed using an exploratory probe. The curing process was performed using a LED light-curing unit set at 1400 mW/cm^2^ (VALO^®^, Ultradent, South Jordan, UT, EUA) for 10 s, 5 s on each side (mesial and distal). The light intensity was measured by a radiometer (Demetron LED radiometer, Kerr Sybron Dental Specialties; Middleton, WI, USA). 

The specimens were numbered and then equally (n=14) and randomly (Random.org Integer Generator; http://www.random.org) divided into five groups (Table 1): C - non-whitening toothpaste (Colgate Tripla Ação, Colgate-Palmolive, SP, Brazil); WTsi - hydrated silica whitening toothpaste (Colgate Optic White, Colgate-Palmolive, NY, USA); WThp - 2% hydrogen peroxide whitening toothpaste (Colgate Luminous White Advanced Expert, Colgate-Palmolive, Gua, Guanajuato, Mexico); OB - in-office bleaching (Whiteness HP 35%, FGM, SC, Brazil); and HB - at-home bleaching (Whiteness Simple 22% FGM, SC, Brazil).

**Table 1:** Testing groups: commercial name, relevant ingredients and manufacturer descriptions.

Group	Commercial name	Relevant ingredients	Manufacturer
C	Colgate Tripla Ação	Sodium monofluorophosphate at 0.76% (0.14% w/v fluoride ion)	Colgate-Palmolive, SP, Brazil
WTsi	Colgate Optic White	Hydrated silica, tetrapotassium pyrophosphate, pentasodium triphosphate	Colgate-Palmolive, NY, USA
WThp	Colgate Luminous White Advanced Expert	Hydrogen peroxide at 2%, sodium monofluorophosphate at 0.76%	Colgate-Palmolive, Gua, Mexico
OB	Whiteness HP 35%	Hydrogen peroxide at 35%	FGM, SC, Brazil
HB	Whiteness Simple 22%	Carbamide peroxide at 22%	FGM, SC, Brazil

Toothbrushing simulations were made using a custom-made automated toothbrushing machine (Biopdi XY, São Carlos/SP, Brazil) with suspensions prepared with the testing toothpastes and distilled water, in a 1:2 ratio, in weigh^11^ ^,^ ^12^ (course: 3.8 cm; velocity: 356 rpm). Soft orthodontic toothbrushes were used (Oral-B Orthodontic Toothbrush 35, P&G, USA). Controlled time, pressure, and temperature (36 ± 1ºC) were applied. The specimens were brushed twice a day for 15 days (500 cycles in each brushing), totalizing 15,000 cycles of circular mechanical brushing. After each brushing cycle, specimens were washed in running water and stored in distilled water at 37ºC.

In group OB, the bleaching agent used was 35% hydrogen peroxide for the in-office procedure. The gel was applied in three applications, each one lasting 15 min. Two bleaching sessions were performed, with a 1-week interval between them. After the bleaching treatment, the teeth surfaces were abundantly washed with water. Between the sessions, the teeth were stored in distilled water at 37ºC, changed daily. 

In group HB, the bleaching agent used was 22% carbamide peroxide for at-home bleaching, for 15 consecutive days, being 2 hours per day. The specimens were stored over cotton gauze soaked with distilled water throughout the bleaching procedure. After the bleaching treatment, the teeth surfaces were abundantly washed with water, and stored in distilled water at 37ºC, changed daily. 

Bracket debonding pliers were used to debond the accessories. Removal of resin remnants was performed using a 12-blade tungsten carbide bur on low-speed handpiece, at 20,000 rpm without water cooling. 

One operator made the color evaluations, using the matrix to standardize the spectrophotometer probe placement during consecutive color evaluations.^14^ ^,^ ^15^ ^,^ ^21^ ^,^ ^22^ The color measurements were recorded at baseline and one month after bracket removal, to give the teeth adequate time to rehydrate.^15^ ^,^ ^23^

The color was measured using color coordinates established by the International Commission on Illumination (CIELab), using the Easyshade device. The following values were evaluated: L*, a*, and b*, in which L* represent the value from 0 (black) to 100 (white), and a* and b* represent the shade, where a* is the measurement along the red-green axis, and b* is the measurement along the yellow-blue axis. The color parameters were measured over a white background. The color comparison before and after treatment was given by the differences between the two shades (ΔE_ab_), which was calculated using the following formula: ΔE_ab_= [(ΔL*)^2^+ (Δa*)^2^+ (Δb*)^2^]^1/2^, where ΔL* = L*(final) - L*(initial); Δa* = a*(final) - a*(initial); and Δb*= b*(final) - b*(initial). To evaluate the perception of tooth whiteness, the CIELab-based whiteness index (WI_D_= 0.511L* −2.324a* −1.100b*) was also calculated. The whiteness index change (ΔWI_D_) was calculated according to the equation: ΔWI_D_ = WI_after bleaching_ - WI_baseline_.^24^ ^,^ ^25^ Three readings were done, and the values were averaged for statistical purposes. The total color alteration (ΔE_ab_) was considered the primary outcome.

During the recording of the tooth color and performing the statistical analyses, the researchers were blinded. 

## STATISTICAL ANALYSES

The data were initially submitted to the Shapiro-Wilk test, and the normality was demonstrated. The compatibility of the baseline CIELab values between the areas compared (experimental - under the bracket; and control - around the bracket) was individually evaluated by paired *t*-test. The paired *t*-test was also applied to the intragroup comparisons between the experimental (under the brackets) and the control (around the brackets) areas, at 1 month after bleaching/whitening procedures. The intergroup comparisons were performed using the one-way ANOVA and Tukey tests. The level of significance adopted was 5%, and statistical analyses were performed using the software Statistica for Windows version 7.0 (Statsoft, Tulsa, Ok, USA).

## RESULTS

The color coordinates evaluated (L*, a*, and b*) were similar at baseline, for all groups, between under the bracket area (experimental) and around the bracket area (control), demonstrating that the areas compared were compatible regarding color at baseline (Table 2).

**Table 2:** Descriptive statistics and intragroup comparisons of CIELab values under the bracket (experimental) and around the bracket (control) areas at baseline.

Initial CIELab	UNDER THE BRACKET AREA (experimental) Mean ± SD	AROUND THE BRACKET AREA (control) Mean ± SD	p
Group C			
L	89.63 ± 2.04	90.82 ± 1.09	0.07
a	1.40 ± 1.19	0.71 ± 0.74	0.09
b	25.1 ± 3.65	22.4 ± 3.89	0.07
Group WTsi			
L	88.8 ± 3.32	90.0 ± 4.41	0.41
a	-1.21 ± 2.13	-2.2 ± 1.81	0.19
b	26.33 ± 6.02	22.68 ± 5.97	0.11
Group WThp			
L	90.17 ± 3.35	91.73 ± 1.92	0.14
a	-2.61 ± 1.61	-2.24 ± 0.90	0.45
b	22.08 ± 3.89	21.68 ± 3.90	0.78
Group OB			
L	88.25 ± 2.81	89.16 ± 3.36	0.44
a	-1.66 ± 0.96	-1.42 ± 1.64	0.64
b	26.27 3.35	25.88 ± 4.53	0.79
Group HB			
L	89.92 ± 2.85	90.91 ± 2.19	0.31
a	1.16 ± 2.00	0.73 ± 1.70	0.55
b	26.39 ± 3.39	24.11 ± 3.06	0.07

SD = standard deviation. Intragroup comparison (paired *t-*test) (α = 0.05). Groups: C = non-whitening toothpaste; WTsi = hydrated silica whitening toothpaste; WThp = 2% hydrogen peroxide whitening toothpaste; OB = in-office bleaching; and HB = at-home bleaching.

» Intragroup comparisons

Intragroup comparisons showed statistically significant differences in color changes (ΔE_ab_) for groups C, WThp and HB, between under and around the bracket areas after bleaching and whitening treatments (group C = 7.97 ± 2.35 x 2.86 ± 0.81, *p*< 0.01; group WThp = 4.69 ± 2.98 x 2.05 ± 1.41, *p*< 0.01; group HB = 7.41 ± 2.89 x 9.86 ± 3.32, *p*= 0.02). In groups C and WThp, the under the bracket area demonstrated significantly greater color change; while in group HB, the around the bracket area underwent significantly greater color change (Table 3).

**Table 3:** Descriptive statistics, intragroup and intergroup comparisons of CIELab and WI_D_ values under the bracket and around the bracket areas, one month after bleaching.

	UNDER THE BRACKET AREA (experimental) Mean ± SD	AROUND THE BRACKET AREA (control) Mean ± SD	p^†^
ΔE_ab_			
C	7.97 ± 2.35^A^	2.86 ± 0.81^A^	< 0.01*
WTsi	8.54 ± 3.63^A^	6.17 ± 4.59^B^	0.08
WThp	4.69 ± 2.98^B^	2.05 ± 1.41^A^	< 0.01*
OB	9.31 ± 4.32^A^	10.72 ± 3.23^C^	0.31
HB	7.41 ± 2.89^A^	9.86 ± 3.32^C^	0.02*
*p*	< 0.01*	< 0.01*	
ΔL*			
C	6.06 ± 2.49^A^	1.08 ± 1.46^AB^	< 0.01*
WTsi	2.78 ± 4.98^B^	3.32 ± 4.27^AC^	0.75
WThp	2.16 ± 3.17^B^	-0.17 ± 1.13^B^	0.02*
OB	5.50 ± 3.85^A^	5.99 ± 2.47^C^	0.65
HB	4.57 ± 2.43^A^	5.30 ± 2.13^C^	0.31
*p*	0.01*	< 0.01*	
Δa*			
C	-2.53 ± 1.16^A^	-1.31 ± 0.72^AB^	< 0.01*
WTsi	-1.65 ± 1.93^AB^	-0.63 ± 1.54^A^	0.12
WThp	-0.68 ± 1.31^B^	-0.18 ± 0.60^A^	0.15
OB	-2.14 ± 0.98^A^	-2.46 ± 1.70^B^	0.50
HB	-2.26 ± 1.08^A^	-1.89 ± 1.34^B^	0.31
*p*	< 0.01*	< 0.01*	
Δb*			
C	-3.75 ± 2.26^AB^	-0.29 ± 1.92^A^	< 0.01*
WTsi	-5.60 ± 4.28^A^	-3.53 ± 3.97^B^	0.26
WThp	-2.43 ± 2.99^B^	-0.74 ± 2.06^A^	0.06
OB	-6.69 ± 3.27^A^	-8.22 ± 2.75^C^	0.16
HB	-4.96 ± 2.48^AB^	-7.78 ± 3.19^C^	< 0.01*
*p*	< 0.01*	< 0.01*	
ΔWI_D_			
C	9.6 ± 4.3^A^	0.2 ± 3.0^A^	< 0.01*
WTsi	7.9 ± 10.9^AB^	3.5 ± 8.2^A^	0.30
WThp	1.8 ± 6.6^B^	-2.6 ± 3.6^A^	0.03*
OB	11.6 ± 6.5^A^	14.3 ± 6.8^B^	0.33
HB	9.4 ± 5.0^A^	12 ± 6.0^B^	0.17
*p*	< 0.01*	< 0.01*	

^†^Intragroup comparison (paired *t*-test). Different superscript uppercase letters in columns indicate statistically significant differences among the groups with ANOVA/Tukey test (α = 0.05). *Statistically significant at *p* < 0.05. Groups: C = non-whitening toothpaste; WTsi = hydrated silica whitening toothpaste; WThp = 2% hydrogen peroxide whitening toothpaste; OB = in-office bleaching; and HB = at-home bleaching.

Lightness (ΔL*) was statistically greater for groups C and WThp at under the bracket area (group C = 6.06 ± 2.49 x 1.08 ± 1.46, *p*< 0.01; group WThp = 2.16 ± 3.17 x -0.17 ± 1.13, *p*= 0.02) (Table 3). 

Group C had, at under the bracket area, statistically significant greater reduction in a* (group C = -2.53 ± 1.16 x -1.31 ± 0.72, *p*< 0.01) and b* (group C = -3.75 ± 2.26 x -0.29 ± 1.92, *p*< 0.01), decreasing redness and yellowness, respectively. Group HB showed a significant reduction in b* (group HB -4.96 ± 2.48 x -7.78 ± 3.19, *p*< 0.01) around the bracket area (Table 3). 

Tooth whiteness (ΔWI_D_) presented statistically significant differences between the areas tested for groups C and WThp (group C = 9.6 ± 4.3 x 0.2 ± 3.0, *p*< 0.01; group WThp = 1.8 ± 6.6 x -2.6 ± 3.6, *p*= 0.03), demonstrating greater alterations for the under the bracket area (Table 3). 

» Intergroup comparisons

Intergroup comparisons demonstrated that the color change (ΔE_ab_) was similar for groups C, WTsi, OB, and HB at under the bracket area (group C = 7.97 ± 2.35; group WTsi = 8.54 ± 3.63; group OB = 9.31 ± 4.32; group HB = 7.41 ± 2.89) (Table 3). Only group WThp showed less color variation than the other groups in this region (group WThp = 4.69 ± 2.98, *p*< 0.01). In the around the bracket area, groups C and WThp showed similar color changes (group C = 2.86 ± 0.81; group WThp = 2.05 ± 1.41), group WTsi demonstrated different color alteration, in comparison with the other groups (group WTsi = 6.17 ± 4.59), and groups OB and HB showed statistically greater color changes (group OB = 10.72 ± 3.23; group HB = 9.86 ± 3.32). 

At under the bracket area, greater lightness (ΔL*) was observed for groups C, OB and HB (group C = 6.06 ± 2.49; group OB = 5.50 ± 3.85; group HB = 4.57 ± 2.43; *p*= 0.01), and at around the bracket area for OB and HB (group OB = 5.99 ± 2.47; group HB = 5.30 ± 2.13; *p*< 0.01). The group WThp demonstrated the smallest lightness change in the control area (group WThp = -0.17 ± 1.13). 

The green-red axis (Δa*) showed greater reduction for groups C, OB and HB at under the bracket area (group C = -2.53 ± 1.16; group OB = -2.14 ± 0.98; group HB = -2.26 ± 1.08; *p*< 0.01); and at around the bracket area for groups OB and HB (group OB = -2.46 ± 1.70; group HB = -1.89 ± 1.34; *p*< 0.01).

At under the bracket area, WTsi and OB demonstrated a statistically significant greater reduction in Δb* (group WTsi = -5.60 ± 4.28; group OB = -6.69 ± 3.27; *p*< 0.01), decreasing yellowness. At around the bracket area, OB and HB showed a greater reduction of yellowness (group OB = -8.22 ± 2.75; group HB = -7.78 ± 3.19; *p*< 0.01) (Table 3). 

Statistically significant greater tooth whiteness (ΔWI_D_) was observed for groups C, OB and HB at the under the bracket area (group C = 9.6 ± 4.3; group OB = 11.6 ± 6.5; group HB = 9.4 ± 5.0; *p*< 0.01), and for groups OB and HB at the around the bracket area (group OB = 14.3 ± 6.8; group HB = 12 ± 6.0; *p*< 0.01).

## DISCUSSION

Although previous studies^14^ ^-^ ^19^ have evaluated the efficacy of different dental bleaching agents under orthodontic brackets, no study has evaluated the whitening toothpastes. Since many patients wearing orthodontic appliances perceive changes in the shade of their teeth, and whitening toothpaste is an easily accessible product,^12^ its efficacy needed to be tested. The null hypothesis was rejected since, considering the parameters ΔE_ab_ and ΔWI_D_, similar results between under and around the bracket areas were observed only for groups WTsi, OB, and HB. 

The efficacy of whitening and bleaching procedures applied in the present study was demonstrated, as observed in the results obtained for the control area (around the bracket). Whitening or bleaching was observed in groups WTsi, OB, and HB by the evaluation method used, and color changes of approximately 6 to 10 ΔE_ab_ were detected. 

Previous studies^26^ ^,^ ^27^ observed that hydrated silica whitening toothpaste showed greater color changes than 2% hydrogen peroxide whitening toothpaste, corroborating with the present results. This finding suggested that the toothbrushing abrasion action was mainly responsible for the color changes.^26^ The authors speculate that the low peroxide hydrogen concentration did not produce enough free radicals to oxidize dentin’s organic component in the WThp group.^28^ ^,^ ^29^ The in-office and at-home bleaching procedures showed statistically greater color changes and similarity around the bracket area, corroborating with previous studies.^18^ ^,^ ^19^ ^,^ ^21^ ^,^ ^22^ ^,^ ^25^ ^,^ ^30^

The area under the bracket showed greater color alteration and tooth whiteness, when compared with the area around the bracket, for the non-whitening toothpaste, 2% hydrogen peroxide whitening toothpaste, and at-home bleaching procedure. Previous studies^31^ ^-^ ^34^ also observed enamel color changes after treatment with fixed orthodontic appliances, demonstrating an increase in the tooth color’s lightness after bracket debonding. The color change under the brackets is variable. Eliades et al.^32^ observed that tooth-color differences after debonding and cleaning procedures ranged from 5.27 ± 2.21 to 13.7 ± 4.7 ΔE_ab_ units. Factors such as acid etching, increased roughness, removal of resin remnants and penetration of resin tags into the enamel surface may have affected the light scattering of the under the bracket area.^32^ ^,^ ^34^ ^-^ ^37^ Sardarian et al^16^ and Lunardi et al^17^ also demonstrated statistically significant lower color change under the bracket than around the bracket, after at-home bleaching procedure. The peroxide concentration (22%) may also have influenced this result.^22^ ^,^ ^28^ ^,^ ^29^

The differences in color change between the areas under and around the brackets were 5.11 units for the non-whitening toothpaste group, 2.37 units for hydrated silica whitening toothpaste group, 2.64 units for 2% hydrogen peroxide whitening toothpaste group, 1.41 units for the in-office bleaching group, and 2.45 units for at-home bleaching group. These results demonstrated that the color change between the areas evaluated was clinically significant only for the non-whitening toothpaste group, since, for the other groups, the ΔE_ab_ values were below the standard value for visual perceptibility and clinical detection of color change (ΔE_ab_ 3.7 units).^38^ Therefore, the color variations probably would not be perceived by the human eye when hydrated silica whitening toothpaste, in-office and at-home bleaching are used in teeth with brackets. 

An average variation of 9.31 and 7.41 of color change (ΔE_ab_) in the area under the bracket for groups OB and HB was observed, which agrees with previous studies:Montenegro-Arana et al^15^ showed a mean variation of 5 to 9 ΔE_ab_; Jadad et al^14^ demonstrated a mean variation of 8.5 ΔE_ab_ after bleaching treatment in teeth with orthodontic brackets; and Sardarian et al^16^ showed a mean variation of 8.51 to 10.84 ΔE_ab_. The absence of previous studies evaluating the effect of whitening toothpaste under orthodontic brackets limits comparisons. 

Despite the limitations of *in vitro* studies, recent studies^11^ ^,^ ^12^ have shown similar results after testing the color variation of whitening toothpastes *in vitro* and *in vivo*. Tao et al^12^ highlighted that the evaluation of tooth whitening products using *in vitro* methods is important for the testing hypotheses and identifying efficacious formulations. Moreover, since this study tested toothpastes, the standardization of toothbrushing procedure was important to eliminate bias. 

Even though the color change was similar under and around the bracket for the bleaching and whitening procedures in groups WTsi and OB, and demonstrated clinical acceptability for HB, it is important to highlight that the use of these products in an abusive manner may result in enamel alterations and tooth sensitivity.^4,15,22,39^ Bleaching is contraindicated in clinical conditions in which the tooth presents exposed dentin or other enamel tissue changes that presumably increase its permeability.^14^

According to the results obtained in the present study, the in-office dental bleaching using 35% hydrogen peroxide, at-home bleaching using 22% carbamide peroxide, and the use of whitening toothpaste containing hydrated silica showed effectiveness in the presence of metallic orthodontic brackets. Therefore, in cases where orthodontic patients request dental bleaching or whitening during orthodontic treatment, these products may be indicated.

Further studies comparing enamel roughness and possible physical and/or mechanical alterations in the orthodontic appliances (e.g. wires, brackets and ligatures) must be encouraged. 

## CONCLUSIONS

Considering the results obtained for the color changes (ΔE_ab_) and the perception of tooth whiteness (WI_D_), the dental color changes were effective, in the presence of metallic orthodontic brackets, for the use of whitening toothpaste containing hydrated silica and in-office dental bleaching with 35% hydrogen peroxide and at-home bleaching with 22% carbamide peroxide.
